# Prenatal affective cognitive training: A proof-of-concept study

**DOI:** 10.1016/j.nsa.2023.101135

**Published:** 2023-09-16

**Authors:** Anne J. Bjertrup, Tue H. Petersen, Nynne Beier, Jeanne Kofoed, Ingeborg Åse Horgen, Anette Kjærbye-Thygesen, Thomas Kirkegaard, Kamilla W. Miskowiak

**Affiliations:** a Copenhagen Affective Disorders Research Center (CADIC), Psychiatric Centre Copenhagen, Mental Health Services, Denmark; b iMotions A/S, Copenhagen, Denmark; c Institute of Clinical Medicine, Faculty of Health and Medical Sciences, University of Copenhagen, Denmark; d Department of Obstetrics and Gynecology, Hvidovre Hospital, Denmark; e Department of Psychology, Faculty of Social Sciences, University of Copenhagen, Denmark

**Keywords:** Cognitive neuropsychiatry, Mother-infant interaction, Emotional cognition, Infant development, Attachment, Pregnancy

## Abstract

Negatively biased cognitive response to infant stimuli during pregnancy is associated with increased postpartum depression (PPD) risk. This proof-of-concept study aimed to investigate the feasibility and effects of a two-week affective cognitive training intervention on cognitive responses to emotional infant stimuli for pregnant participants at risk of PPD. Forty-three participants were included: 23 ​at high risk and 22 ​at low risk of PPD. Cognitive response to emotional infant stimuli was assessed at baseline, immediately after the intervention and at a two-week follow-up for the intervention group (n ​= ​16) and twice over two weeks for the comparison group. The intervention was feasible, as 80% completed all sessions and gave positive feedback. The intervention group showed increased sensitivity to happy infant faces (p-values < 0.02, d ​> ​0.1), more infant-directed facial expressions (p ​< ​0.001, d ​= ​0.6), greater attention toward infant stimuli (p ​= ​0.04, d ​= ​0.2), and reduced negative reactivity to infant distress (p ​= ​0.01, d ​= ​2.6). The increased sensitivity to happy infant faces correlated with fewer depressive symptoms six months after birth (r = −0.59, p = 0.03). Interpretation of the results is limited by the lack of a high-risk control group and small sample size. Randomised controlled trials are now warranted to investigate whether the effects of prenatal affective cognitive training on affective cognitive response to infant stimuli confer reduced risk of PPD.

## Highlights

• This is the first study of affective cognitive intervention for pregnant women at high-risk of postpartum depression.
• Computerised affective cognitive training for pregnant women at risk of postpartum depression is feasible and well-tolerated.
• Cognitive processing of and reactions to emotional infant stimuli can be modified with computerised training paradigms.

## 1 Introduction

Negative cognitive bias has been shown to constitue a risk- and maintaining factor for depression (Penton-Voak et al., 2017; Gotlib et al., 2004). Emerging evidence indicates that negatively biased cognitive processing of *infant* signals during pregnancy marks an increased risk of postpartum depression (PPD) even for people with no depression history (Bjertrup et al., 2021a). This is in line with a key assumption in cognitive neuropsychiatry that abnormalities in cognitive processes underlie psychopathology (Halligan et al., 2001). Although, the multicausal mechanisms in PPD remain elusive, targeting such abnormal affective cognitive processes could be a mechanistically essential tool for reducing PPD risk.

Mothers with PPD gaze less at their infants, display more negative facial expressions, decreased imitation, fewer expressions signalling comfort, and excitement during mother-infant interactions (Cohn et al., 1990; Field et al., 2003, 2005; Mavrogiorgou et al., 2022) and have difficulties with regulating their own and their infants' emotions (Riva Crugnola et al., 2016). We showed that *asymptomatic* mothers with recurrent depression displayed increased visual attention toward sad infant faces on a computerised task but avoided looking at videos of intense infant happiness and distress (Bjertrup et al., 2021b). These mothers also displayed more negative facial expressions when listening to infant cries than mothers with no psychiatric history, suggesting that they were more negatively affected (Bjertrup et al., 2021b). Such negatively biased maternal responses can limit infants' acquisition of emotion regulation abilities and experiences of positive emotions (Feldman, 2007; Field, 2010) and thereby mediate the adverse effects of PPD on child development. Indeed, maternal PPD increases offspring risk of socio-emotional developmental delays at age two (Chorbadjian et al., 2020), anxiety at age 10 (Morales-Munoz et al., 2022) and depression at age 24 years (Culpin et al., 2021). The association between PPD and depression in offspring was partly mediated by negative maternal perceptions of and responses to infant crying (Culpin et al., 2021).

Some evidence suggests that computerised attentional bias modification reduces negative cognitive bias in depression (Woolridge et al., 2021; Vazquez et al., 2016), and that cognitive bias modification increases healthy mothers’ sensitivity to infant happiness (Carnegie et al., 2016). Further, mindfulness-inspired emotion regulation training may enhance mothers' ability to remain calm and display well-regulated and sensitive maternal behaviour (Potharst et al., 2017). Finally, working memory training has been shown to improve emotion regulation abilities across psychiatric diagnoses (Barkus, 2020). Notwithstanding the emerging evidence for beneficial effects of these affective cognitive training approaches, there is no published study of whether targeting affective cognitive biases and emotion regulation ability during pregnancy can modulate negative biases or reduce PPD risk.

Following a cognitive neuropsychiatric approach, this study aimed to investigate the feasibility and effects of a novel multilevel affective cognitive training intervention targeting attention, interpretation, emotion regulation abilities and emotional biases in pregnant participants at high-risk of PPD, here defined as a history of previous depression. Our intervention aimed to guide high-risk mothers in responding less negatively and more balanced and well-regulated to infant distress signals, recognizing the benefits of some emotional and psychophysiological reactivity to infants' needs. Excessive negative reactivity to aversive stimuli is a risk factor for PPD (Bjertrup et al., 2021a, 2023). Thus, our objective was to shift mothers' reactions from excessive frustration and negative evaluations to more empathic and positive responses to infants' emotions and vocalizations. We hypothesised that the affective cognitive intervention would be well tolerated for high-risk pregnant participants, as reflected by low drop-out and positive participant feedback. We further hypothesised that, after the intervention, they would show (I) reduced negatively biased perception of infant stimuli/increased sensitivity to infant happiness, (II) reduced negative emotional reactivity toward distressed infant stimuli and (III) enhanced infant-directedness in facial expressions and attention.

## 2 Material and methods

### 2.1 Participants and screening

Participants were included and affective cognitive assessments and training sessions took place from March 2021 until January 2022. The postpartum follow-up assessment of depressive symptoms was assessed with an online questionnaire between January 2022 and June 2022. Pregnant high-risk participants were recruited from the Family Clinic at Hvidovre Hospital and The Copenhagen Affective Disorder Clinic. Pregnant low-risk participants were recruited from the department of obstetrics and gynaecology, Hvidovre and Amager Hospital. Participants were also recruited from online advertisements on Moedrehjælpen.dk and Forsoegsperson. dk. General inclusion criteria were age≥18 years and sufficient Danish language skills. Participants were screened with the Mini International Neuropsychiatric Interview (MINI) (Sheehan et al., 1998), the Hamilton Depression Rating Scale-17 items (HDRS-17) (Hamilton, 1960) and the Standardised Assessment of Personality–Abbreviated Scale (SAPAS) (Hesse et al., 2010). Inclusion criteria for high-risk pregnant participants were one or more previous episodes of depression and/or (hypo)mania formally diagnosed by a clinician and confirmed with MINI in this study. Exclusion criteria were severe depression (HDRS-17 ​≥ ​17) with onset in the current pregnancy, ADHD, borderline personality disorder, schizoaffective disorder, neurological illness, alcohol, or substance abuse (defined by ICD-10 F10.1 or F10.2 criteria). Inclusion criteria for low-risk pregnant participants were no personal history of mental illness, no indication of personality disorder (SAPAS<3), and no first-degree family history of depression, BD, or schizophrenia. All participants gave written informed consent, and the study was conducted in accordance with the Declaration of Helsinki (version 2013). The study was approved by the Danish Data Protection Agency Capital Region of Denmark (ID P-2021-199) and by the local ethics committee in the Capital Region of Denmark (ID H-21000595).

### 2.2 Procedures

**Fig. 1:**
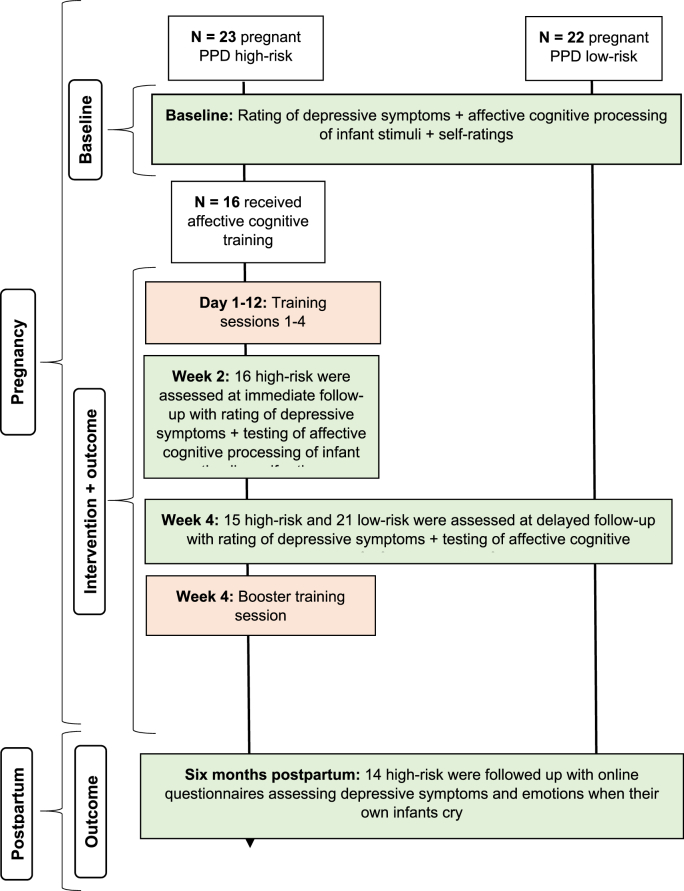
Flow chart displaying study design and number of included participants. The time points stated for each assessment are median test, training, and follow-up times for participants. One high-risk lost to postpartum follow-up due to still birth.

At inclusion, we obtained demographic and clinical information. The baseline visit had a duration of 1.5 ​hours and included ratings of depressive symptoms with the HDRS-17, computerised affective cognitive processing of infant stimuli and questionnaires. The high-risk pregnant participants went through four affective cognitive 1 hour training sessions over 8–27 days (median: 12 days). Each training session included depression symptom rating and five computerised training tasks aiming to improve (1) visual attention toward infants, (2) infant-directedness in facial expressions and attention, (3) recognition of infant happiness, and (4) implicit and (5) explicit emotion regulation strategies in response to infant distress. Affective cognitive processing of infant stimuli was investigated using the same computer tasks employed at baseline immediately after the fourth training session and again after approximately two weeks (median: 14 days; interval: 10–35 days). This timing of the second follow-up enabled investigation of whether any affective cognitive changes would prevail beyond the acute treatment phase while minimising the risk that birth would take place before the assessment. Immediately after this second assessment, high-risk pregnant received a booster training session to enhance the potential effect of the training. The low-risk pregnant participants did not undergo any intervention but were re-assessed with the computerised tasks after approximately two weeks (median: 14 days; interval 11–21 days). This enabled investigation of the test-retest reliability of affective cognitive processing of infant stimuli. Six months after birth (median: 6.5 months), participants filled out an online questionnaire assessing depressive symptoms. See Fig. 1 for at flow-chart displaying assessment timepoints and number of participants included at each stage.

### 2.3 Materials

Affective cognitive tasks and training paradigms were run on a Lenovo T430 14″ laptop with 1920 ​× ​1080 resolution monitor using iMotions Software version 9 (iMotions), Psychtoolbox in MATLAB with custom-made scripts or E-prime version 2 (Tools, 2013). For infant vocalisation and video stimuli, audio was played through headphones. While participants carried out computerised tasks, their eye gazes and fixations, electrodermal activity (EDA) and facial expressions were recorded using a Tobii eye-tracker, EDA-finger-sensors and the computer's web camera from which facial Action Units (AU's) displayed by participants were continuously analysed with the Affectiva AFFDEX 4.0 algorithm (McDuff et al., 2016). We analysed facial expression valence and an “infant-directedness” composite consisting of the AUs comprising mothers' comforting and caring facial expressions directed toward their infants (Chong et al., 2003) (see Supplementary for details).

### 2.4 Affective cognitive assessment battery

#### 2.4.1 Infant emotional rating task

Pregnant participants rated 50 infant face images and 50 infant vocalisations. Infant face images (same stimuli as in Parsons et al., 2019) (Parsons et al., 2019) and infant vocalisations (from the OxVoc database (Parsons et al., 2014)), in five different emotional categories (most happy, moderately happy, neutral, moderately distressed and most distressed) were presented for 2 ​seconds. Within 5 ​seconds, participants indicated on a rating-bar (1) how happy or sad the infant was, and (2) their own (dis)comfort (for vocalisation only). Participants went through a short practice round with images of neutral objects (shoes) to familiarise themselves with the stimulus presentations and the rating bar. Total task duration was 25 ​minutes.

#### 2.4.2 Attention to emotional infant faces

The infant dot-probe task started with a 500 milliseconds (ms) fixation cross, followed by a 500 ms presentation of infant-infant or infant-adult face-image-pairs displaying distressed, happy, or neutral (infants only) expressions in pseudo-random order so pairs from the same condition were not repeated twice in a row. Then two small dots appeared, replacing one of the images an equal number of times for each face in a pair. Participants were instructed to indicate whether the dots were vertical (-) or horizontal (··) by pressing one of two labelled keys as fast as possible. The dots remained on the screen until a key was pressed. Faster correct responses to dots that appeared in the previous location of a specific face indicate an attentional bias toward that face. A total of 160 presentations of face-pairs were comprised by four conditions: (i) 40 distressed vs. neutral infant faces, (ii) 40 happy infant vs. neutral infant faces, (iii) 40 distressed infant vs. distressed adult faces and (iv) 40 happy infant vs. happy adult faces. We calculated four “vigilance” variables by subtracting accuracy/response times (RT) for adult or neutral infant targets from accuracy/RT for infant emotional targets, so higher scores reflected greater vigilance toward infant emotional targets. Infant and adult emotional faces were grey scale images from Kringelbach, Lehtonen (Kringelbach et al., 2008) and Ekman and Friesen (Ekman et al., 1976), respectively. The duration was 10 ​minutes.

#### 2.4.3 Emotional responses to infant distress videos

While watching a 28 ​second video displaying an infant crying heavily without being attended to, participants were instructed to imagine that the infant was theirs. After the video, participants rated their own emotional reactions to the crying infant with the “mother-oriented frustration” scale (hereinafter *frustration*) from “The My Emotions Questionnaire” (Leerkes et al., 2020). Task duration was 10 ​minutes.

### 2.5 Questionnaires and ratings

At baseline, participants filled out the questionnaires Edinburgh Postnatal Depression Scale (EPDS) (Smith-Nielsen et al., 2018), State and Trait Anxiety Inventory (STAI-S and -T) (Spielberger, 1983), Perceived Stress, Visual Analogue Scales (VAS) assessing various symptoms such as nausea and fatigue, and the Maternal Antenatal Attachment Scale (MAAS) (Maas et al., 2016). The VAS and STAI-S questionnaires were re-administered at immediate and delayed follow-up to explore changes in subjective state from before to after the intervention that might influence affective cognition. The EPDS was readministered online for the high-risk intervention group six months after birth.

### 2.6 Affective cognitive training

Pregnant high-risk participants in the intervention group went through five training tasks each with a duration of 10 ​min. In the *attentional bias modification task*, they watched a block of 10 videos of infants crying and laughing - one at a time - and were instructed to look at infants’ faces. Real-time information from the eye-tracker on gaze behaviour fed back into the task and manipulated the visual stimuli, so that the screen got blurry and red if they gazed away from the infant. Videos had a duration of 10–28 ​seconds and were shown in random order in a block that was repeated twice.

In the *infant-directed facial expression task*, participants were instructed to use their facial expressions to *empathise with* and *mirror* the emotional states displayed by infants in nine videos of three different emotional categories: distressed, happy, and calm. The AUs of facial expressions in response to each video were continuously analysed and estimated against predefined criteria for correct infant-directed facial expressions (Chong et al., 2003) and the participants received feedback. The computer chose one of several predefined feedback texts randomly (see Supplementary for details on AUs). A block of videos consisted of nine videos (three in each category) shown in a randomised order and was repeated once, so participants could incorporate feedback in their facial expressions when they interacted with the infant video the second time.

The *cognitive bias modification task* consisted of grey scale images of 50 emotional infant faces (from Kringelbach, Lehtonen (Kringelbach et al., 2008)) in five different categories (very happy, moderately happy, very distressed, moderately distressed and ambiguous/neutral faces) presented for 2 ​s on the screen in random order. High-risk pregnant participants were asked to evaluate whether the infant was happy or sad and reply as fast as possible. They received feedback before the next image appeared: congruent for the happy and distressed faces, but for the 10 neutral infant faces, they were told that the infant was happy.

In an *implicit emotion regulation task,* participants completed the verbal letter-variant N-back task, while listening to infant cries. They were asked to press a key, whenever the stimulus letter matched the one from N (1, 2, or 3) trials back in the letter sequence. Consonants were shown in varying cases: b, B, d, D, p, P, t, T, v, V. The task difficulty increased with three cognitive load levels; first the 1-back, then 2-back and finally the 3-back condition presented in four blocks in a pseudo random order. Each block consisted of 10 letter trials of which three were targets and each letter was displayed for 0.5 ​seconds. Letters in a block were interleaved by a 1.5 ​second fixation cross. The infant cry vocalisations were obtained from Parsons, Young (Parsons et al., 2014).

In an *explicit emotion regulation task*, participants received written instructions on how to remain calm and attentive toward five 30 second infant cry videos. The task ended with a visualisation practice, where participants closed their eyes, and turned their attention toward their own infant (see Supplementary for instructions). The videos and instructions were presented in a fixed order, and they were repeated once.

### 2.7 Statistics

The effect of the affective cognitive training was analysed using Linear Mixed Models (LMM) with restricted maximum likelihood as method of estimation that uses the full, though incomplete, data set and with time (baseline, immediate follow-up, and delayed follow-up), emotion (i.e., emotion of infant face images, vocalizations and videos or identity of dot-probe targets) and time∗emotion as fixed factors. Affective cognitive measures (i.e., ratings, facial expressions, gazes, fixations, frustration, EDA, RT, and accuracy) were dependent variables in 18 separate LMM analyses. For significant primary LMM analyses, we investigated if the results were driven by significant changes from baseline to immediate follow-up and/or baseline to delayed follow-up. For significant interactions, we explored the changes for each emotion/level separately and reported parameter estimates and p-values for significant emotion/levels of the independent variable. Significant affective cognitive changes in the intervention group were correlated with depressive symptoms after birth.

Baseline affective cognitive differences between groups were investigated with repeated measures analysis-of-variance (ANOVA) with affective cognitive score as within-subjects factor and group as between-subjects factor. Significant interactions were followed up by t-tests for data distributions that met Shapiro-Wilk test criteria for normality and by Mann-Whitney U tests for non-normally distributed data. Primary analyses were adjusted for group differences in depressive symptoms by including these as covariates in post hoc analyses. Group differences on demographic and clinical variables were analysed with one-way ANOVA. Normative changes and test-retest reliability of affective cognitive assessments from baseline of follow-up were investigated for the low-risk pregnant participants with LMM and correlation analyses (Pearson coefficient r or Spearman's Rho for normally or non-normally distributed data, respectively), respectively. Tests were two-tailed with a significance level of 0.05 and effect sizes for significant results on neurocognitive tasks were reported as Cohen's d, partial eta squared η^2^, or Pearson correlation coefficient r depending on the analysis (LMM, *t*-test, ANOVA or Mann-Whitney U). Post hoc adjustment for multiple comparisons was conducted with the Benjamini-Hochberg (B–H) procedure with a false discovery rate of 0.05. Data were analysed with the Statistical Package for the Social Sciences (SPSS) version 25.

## 3 Results

### 3.1 Demographics and clinical characteristics

Twenty-three high-risk pregnant participants between 23 and 39 weeks gestation and 22 low-risk pregnant participants between 22 and 38 weeks gestation were included. Three high-risk pregnant only wanted to participate in the baseline assessment because of long transportation time to the research centre. Of the 20 high-risk pregnant included in the intervention, two dropped out due to family reasons, one gave birth prematurely and one had severe pelvic pain. Sixteen (80%) high-risk pregnant participants completed the intervention and the immediate follow-up, while 15 (75%) completed both the immediate and delayed follow-up. Twenty-one (95%) of the low-risk pregnant participants were followed-up. Two high-risk participants were lost to post-partum follow-up; one had a still birth, and one did not reply to the questionnaires. Thus, 14 high-risk were followed up six months after birth.

Around half of the high-risk pregnant participants took psychotropic medication, while low-risk pregnant were medication free (Table 1). There were no differences in age or years of education (p-values≥0.23) or parity, number of primiparous participants or gestational age at baseline (p-values ≥0.10). At baseline, high-risk pregnant experienced more depression symptoms than low-risk pregnant (HDRS-17: F(1,43) ​= ​9.63, p ​= ​0.003; EPDS: F(1,43) ​= ​16.26, p ​≤ ​0.001), and had more dysfunctional personality traits (F(1,43) ​= ​36.42, p ​≤ ​0.001) and trait anxiety than low-risk pregnant (F(1,43) ​= ​5.72, p ​= ​0.02). Quality of maternal antenatal attachment was lower among high-risk than low-risk pregnant participants (F(1,42) ​= ​6.96, p ​= ​0.01), while there were no differences in intensity of or total antenatal attachment (p-values≥33). High-risk pregnant experienced more sadness (F(1, 43) ​= ​14.65, p ​≤ ​0.001), anxiety (F(1, 43) ​= ​13.17, p ​≤ ​0.001), dizziness (F(1, 43) ​= ​10.60, p ​= ​0.002), unpleasantness (F(1, 43) ​= ​20.34, p ​≤ ​0.001), and less happiness (F(1, 43) ​= ​4.60, p ​= ​0.04) at baseline than low-risk pregnant (see Supplementary Table A**)**. At immediate follow-up, high-risk pregnant experienced more depression symptoms and anxiety and dizziness than low-risk pregnant (HDRS-17: F(1,35) ​= ​12.12, p ​= ​0.001; VAS anxiety (F(1, 36) ​= ​4.98, p ​= ​0.03); dizzy: (F(1, 36) ​= ​12.50, p ​= ​0.001), but there was no difference in state anxiety (p ​= ​0.29).

**Table 1:** Demographics, clinical characteristics, affective symptoms, attachment to the unborn child and affective cognition at baseline in high-risk pregnant and low-risk pregnant participants.

	Baseline scores, mean (SD)		*p*-value group	*p*-value interaction
	High-risk pregnant (N ​= ​23)	Low-risk pregnant (N ​= ​22)		
Age in years	31.1 (4.1)	30.5 (4.1)	0.61	
Years of education	15.7 (1.6)	16.3 (1.6)	0.23	
Primiparous, n (%)	15.0 (65.2)	18 (81.9)	0.22	
Parity, median (range)	0 (0–1)	0 (0–3)	0.32	
GA baseline	30.4 (3.6)	32.3 (3.9)	0.10	
GA T1	31.4 (3.6)	34.3 (3.8)	0.03	
GA T2	33.5 (3.6)			
EPDS baseline	7.8 (5.2)	2.8 (2.8)	≤0.001	
EPDS six months postpartum follow-up	6.1 (3.7)			
HDRS baseline	5.5 (5.2)	1.8 (2.1)	0.003	
HDRS T1	5.6 (6.0)	1.0 (1.4)	0.001	
HDRS T2	4.1 (4.3)			
STAI-S baseline	47.7 (5.4)	49.2 (4.3)	0.52	
STAI-S T1	45.5 (4.4)	46.9 (3.0)	0.29	
STAI-S T2	45.6 (5.8)			
STAI-T baseline	48.0 (6.2)	44.9 (3.4)	0.02	
SAPAS	3.0 (1.5)	0.8 (0.8)	≤0.001	
Age of depression onset, years	22.8 (6.2)			
Number of depressive episodes, median (range)	2.0 (3.0)			
Psychotropic medication, n (%)	12.0 (52.2)	1 (4.5)	≤0.001	
Antidepressant, n (%)	10 (43.5)	0 (0)		
Antipsychotic, n (%)	0 (0)	0 (0)		
Anticonvulsive, n (%)	2 (8.7)	1 (4.5)		
MAAS total	77.0 (7.0) ^a^	78.9 (5.4)	0.33	
MAAS quality	44.0 (3.1)	46.3 (2.5)	0.01	
MAAS intensity	29.0 (3.9)	28.5 (3.8)	0.67	
Infant face images, rating	0.03 (0.21)	0.02 (0.31)	0.93	0.53
Infant vocalisations, rating	−0.26 (0.30)	−0.30 (0.40)	0.67	0.68
Emotional reactivity to infant vocalisations	−0.07 (0.45)	0.01 (0.56)	0.64	0.58
Frustration to infant distress video	1.39 (0.55)	1.26 (0.31)	0.34	
Electrodermal activity peaks, infant distress video	4.19 (2.04) ^b^	4.29 (2.26)	0.89	
Facial expression to infant vocalisations, valence	−0.82 (2.62)	−2.09 (4.12)	0.24	0.53
Facial expression to infant images, valence	−0.90 (4.98) ^b^	0.26 (5.65)	0.47	0.18
Facial expression to infant distress video, valence	−0.50 (9.05)	0.35 (7.37)	0.73	
Accuracy infant vs adult	0.04 (0.45)	0.28 (0.80) ^c^	0.24	0.046
Accuracy infant emotion vs infant neutral	0.48 (0.99)	0.55 (1.01) ^c^	0.82	0.78
RT vigilance infant vs adult	27.91 (32.38)	28.54 (58.02) ^c^	0.96	0.42
RT vigilance infant emotion vs infant neutral	−12.85 (44.01)	−3.84 (44.26) ^c^	0.51	0.15
Gaze toward infant images	92.61 (8.32) ^b^	93.64 (4.76) ^c^	0.53	0.65
Fixations toward infant images	96.23 (4.38) ^b^	95.87 (4.13) ^c^	0.80	0.27
Gaze toward infant distress video	90.88 (5.29)	88.05 (11.21) ^c^	0.29	0.22
Fixations toward infant distress video	94.48 (4.08)	92.32 (9.33) ^c^	0.32	0.46
Facial expression infant images, infant-directed	0.95 (1.22) ^b^	1.59 (2.02)	0.21	0.48
Facial expression to infant video, infant-directed	1.81 (2.86)	2.50 (3.18)	0.45	

Abbreviations: GA, gestational age; HDRS, Hamilton Depression Rating Scale; EPDS, Edinburgh Postnatal Depression Scale; STAI-S and T, State-Trait Anxiety Inventory; SAPAS, Standardised Assessment of Personality – Abbreviated Scale; MAAS, Maternal Antenatal Attachment Scale. p –values for main effect of group or group∗emotion interaction. ^a^ Due to experimenter error answers are missing on the MAAS for one high-risk pregnant woman. ^b^ Due to technical issues, two high risk pregnant have missing EDA data and gaze, fixation, and facial expression data for infant images. ^c^ Three low-risk pregnant participants have missing gaze and fixation data for infant images, two low-risk have missing dot-probe data and one low-risk woman has missing gaze and fixation data for the infant video. Data are missing completely at random, why the missing data do not introduce bias in the results (Donders et al., 2006). We did not replace missing data points with imputation methods and therefore, analyses of the tasks with missing data were run with fewer data points.

### 3.2 Baseline affective cognition

An interaction effect on the dot-probe test (F(1,41) ​= ​4.24, p ​= ​0.046, η^2^ ​= ​0.09, B–H adjusted p ​= ​0.37), was driven by a trend to lower accuracy for infant vs. adult happy faces in high-risk than low-risk pregnant participants (U ​= ​158.0, p ​= ​0.06, r ​= ​0.29). This trend disappeared after controlling for HDRS-17 score (p-values≥0.13). There were no other baseline group differences in affective cognition (p-values≥0.15).

### 3.3 Feasibility

No adverse effects of the intervention were reported, and the reasons for the four dropouts were not related to the intervention but pregnancy-related factors. In general, participants replied that the explicit emotion regulation and infant-directed facial expression task improved their present-moment awareness and their sense of self-worth regarding motherhood, respectively. A few participants reported that it was difficult for them to stay focused and uncomfortable to interact with an infant on the computer screen. Of the participants who gave feedback on the intervention, half replied that they thought the intervention would reduce their risk of PPD.

### 3.4 Does the intervention reduce negatively biased perception of infant stimuli and increase sensitivity for infant happiness?

**Fig. 2:**
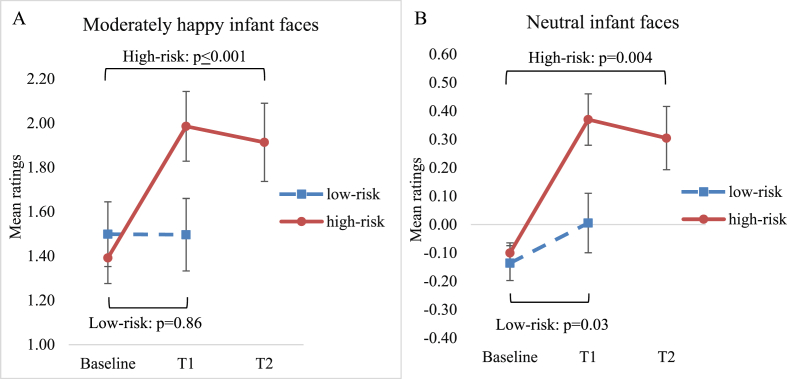
High-risk pregnant women showed increased sensitivity in the perception of infant happiness at immediate follow-up (T1) and delayed follow-up (T2) compared to baseline (red solid line) for both (A) happy infant faces and (B) neutral infant faces. For low-risk pregnant women, responses remained stable for the happy infant faces (A), while they rated neutral infant faces (B) slightly more positive at T1 (blue dotted line). Error-bars represent standard error of the mean. (For interpretation of the references to colour in this figure legend, the reader is referred to the Web version of this article.)

On the infant emotion rating task, participants showed enhanced sensitivity toward happy infant facial expressions over time (F(2,74.53) ​= ​13.34, p ​≤ ​0.001, B–H adjusted p ​≤ ​0.001); interaction: (F(8,74.53) ​= ​6.14, p ​≤ ​0.001; B–H adjusted p ​≤ ​0.001). These effects were driven by more positive ratings of happy and neutral infant face images at immediate and delayed follow-up than baseline (Table 2 and Fig. 2). There was an overall effect of time on ratings of infant vocalisations (F(2,75.04) ​= ​4.38, p ​= ​0.02; B–H adjusted p ​= ​0.01; interaction: p ​= ​0.23). Separate changes from baseline to immediate or delayed follow-up were not significant (p-values≥0.14).

**Table 2:** Perception of infant emotions in high-risk pregnant participants at baseline, immediate follow-up (N ​= ​16) and delayed (N ​= ​15) follow-up.

	T0 Mean (SD)	T1 Mean (SD)	T2 Mean (SD)	T0 – T1				T0-T2			
				*F*-value (*df*_1_, *df*_2_)/Parameter estimate [95% CI]	*p*-value time	*p-*value interact.	d	*F*-value (*df*_1_, *df*_2_)/Parameter estimate [95% CI]	*p*-value time	*p-*value interact.	d
*Ratings of infant faces*											
Across all emotions	0.0 (1.8)	0.2 (2.1)	0.3 (0.5)	26.36 (1,79.27)	≤0.001	≤0.001	0.1	6.24 (1,70.96)	0.02	≤0.001	0.2
Most happy	2.5 (0.6)	2.8 (0.6)	2.8 (0.6)	0.25 [0.03, 0.47]	0.03		0.5	0.12 [0.16, 0.49]	0.39		
Moderately happy	1.4 (0.6)	2.0 (0.7)	1.9 (0.7)	0.65 [0.46, 0.85]	≤0.001		0.9	0.52 [0.34, 0.67]	≤0.001		0.8
Neutral	−0.1 (0.2)	0.4 (0.4)	0.3 (0.5)	0.47 [0.28, 0.66]	≤0.001		1.1	0.40 [0.15, 0.66]	0.004		1.1
Moderately distressed	−1.3 (0.4)	−1.5 (0.5)	−1.5 (0.6)	0.13 [-0.10, 0.36]	0.24			0.16 [-0.13, 0.45]	0.25		
Most distressed	−2.4 (0.5)	−2.5 (0.5)	−2.6 (0.6)	0.10 [-0.10, 0.30]	0.32			0.20 [-0.11, 0.51]	0.19		
*Ratings of vocalizations*											
Across all emotions	−0.3 (0.3)	−0.2 (0.2)	−0.3 (0.3) ^b^	2.21 (1, 81.43)	0.14	0.30		0.14 (1, 75.30)	0.71	0.14	

Abbreviations: T0, baseline; T1, immediate outcome; T2, delayed outcome; CI, confidence interval; df_1_, numerator degrees of freedom; df_2_, denominator degrees of freedom; interact., interaction; *F*-values are given for the effect of time. Significant interaction effects are followed by parameter estimates for the specific levels of emotion. *p*-values are listed for the main effect of time (*p*-value time) and for the emotion∗time interaction effect (*p*-value interaction). Cohens *d* specify the effect size for significant changes from T0 to T1 and from T0 to T2.

### 3.5 Does the intervention reduce emotional reactivity to distressed infant stimuli?

**Fig. 3:**
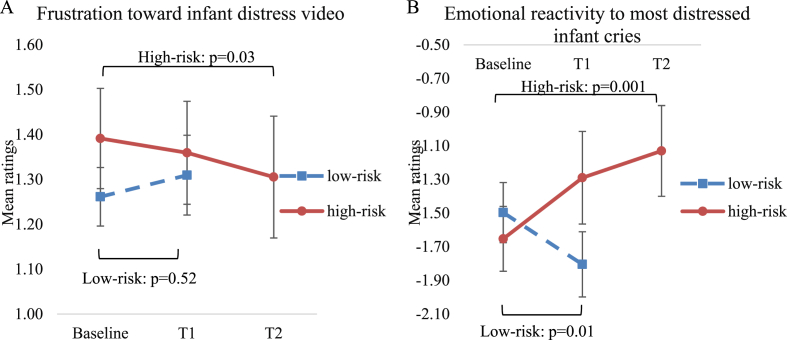
(A) High-risk pregnant women showed decreased frustration in response to the infant distress video at immediate follow-up (T1) and delayed follow-up (T2) compared to baseline (red solid line), while responses from low-risk pregnant women remained stable (blue dotted line). (B) High-risk pregnant women rated their own emotions in response to the most distressed infant cries as less negative at immediate follow-up (T1) and delayed follow-up (T2) compared to baseline (red solid line). Low-risk pregnant women rated their own emotions as more negative at T1 (blue dotted line). Error-bars represent standard error of the mean. (For interpretation of the references to colour in this figure legend, the reader is referred to the Web version of this article.)

In response to the infant distress video, participant's frustration was reduced over time (F(2,17.03) ​= ​3.64, p ​= ​0.048, B–H adjusted p ​= ​0.10). This was driven by a reduction from baseline to delayed follow (Table 3 and Fig. 3A). Participants showed a non-significant trend toward decrease in EDA over time to the distressed infant video (F(2,15.46) ​= ​3.57, p ​= ​0.053, B–H adjusted p ​= ​0.08), driven by significant decrease from baseline to immediate and baseline to delayed follow-up assessments.

**Table 3:** Emotional reactivity toward distressed infant stimuli in high-risk pregnant participants at baseline, immediate follow-up (N ​= ​16) and delayed (N ​= ​15) follow-up.

	T0 Mean (SD)	T1 Mean (SD)	T2 Mean (SD)	T0 – T1				T0-T2			
				*F*-value (*df*_1_, *df*_2_)/Parameter estimate [95% CI]	*p*-value time	*p-*value interact.	d	*F*-value (*df*_1_, *df*_2_)/Parameter estimate [95% CI]	*p*-value time	*p-*value interact.	d
*Responses to infant distress video*											
Frustration	1.4 (0.6)	1.4 (0.5)	1.3 (0.5)	1.62 (1, 18.82)	0.22			5.59 (1,16.25)	0.03		0.2
Electrodermal activity peaks	4.2 (2.0)	2.6 (2.1) ^a^	2.3 (1.9) ^b^	6.37 (1,17.42)	0.02		0.8	5.97 (1,16.26)	0.03		1.0
Facial expression valence	−0.5 (9.1)	−1.6 (3.2)	−1.3 (11.9)	0.42 (1, 20.37)	0.53			0.07 (1, 15.74)	0.80		
*Response to infant face stimuli*											
Facial expression valence	−0.9 (5.5)	−2.9 (5.1)	−2.0 (9.3)	7.54 (1, 85.23)	0.01	0.62	0.4	0.92 (1, 69.92)	0.34	0.08	
*Response to infant vocalizations*											
Facial expression valence	−0.8 (3.5)	−1.5 (4.0)	−1.1 (3.1)	1.36 (1, 56.08)	0.25	0.88		3.02 (1, 52.32)	0.09	0.71	
*Rating of own emotions in response to infant vocalizations*											
Across all emotions	−0.1 (1.4)	0.0 (1.4)	0.0 (1.2)	1.12 (1, 76.02)	0.29	0.33		7.70 (4, 75.13)∗	0.90	≤0.001	0.1
Most happy	1.3 (0.9)	1.3 (1.1)	1.1 (0.9)	0.10 [0.75, 1.72]	0.57			−0.30 [-0.64, 0.03]	0.07		
Moderately happy	1.0 (0.7)	0.9 (0.8)	0.6 (0.5)	0.03 [-0.20, 0.27]	0.76			−0.39 [-0.61,-0.17]	0.001		0.7
Neutral	0.2 (0.5)	0.3 (0.3)	0.3 (0.4)	−0.06 [-0.22, 0.10]	0.45			0.002 [-0.16, 0.16]	0.98		
Moderately distressed	−1.2 (0.8)	−1.0 (1.0)	−1.1 (0.8)	−0.16 [-0.50, 0.18]	0.33			0.24 [-0.02, 0.51]	0.07		
Most distressed	−1.7 (1.0)	−1.3 (1.1)	0.3 (0.4)	−0.24 [-0.51, 0.03]	0.07			0.37 [0.11, 0.63]	0.01		2.6

Abbreviations: T0, baseline; T1, immediate outcome; T2, delayed outcome; CI, confidence interval; df_1_, numerator degrees of freedom; df_2_, denominator degrees of freedom; interact., interaction; Acc., accuracy; emo., emotion; RT, response time; express, expression. *F*-values are given for the effect of time. ∗for the T0-T2 change in *emotional reactivity to infant vocalizations*, the *F*-value is reported for the interaction effect. Significant interaction effects are followed by parameter estimates for the specific levels of emotion. *p*-values are listed for the main effect of time (*p*-value time) and for the emotion∗time interaction effect (*p*-value interaction). Cohens *d* specify the effect size for significant changes from T0 to T1 and from T0 to T2. ^a^ Due to technical issues, EDA data for infant distress video are missing for two high-risk at T1. ^b^ At T2, EDA are missing for three high-risk pregnant. Data are missing completely at random, why the missing data do not introduce bias in the results (Donders et al., 2006). We did not replace missing data points with imputation methods and therefore, analyses of the tasks with missing data were run with fewer data points.

In response to infant vocalizations, participants experienced *fewer negative* emotions to the most distressed infant cries but *less positive* emotions to moderately happy vocalizations at delayed follow-up than baseline (interaction: F(8,75.10) ​= ​4.00, p ​= ​0.001, B–H adjusted p ​= ​0.003; time: p ​= ​0.35) (Table 3 and Fig. 3B).

In response to infant face images, participants displayed more *negative* facial expressions over time (time: F(2,70.46) ​= ​3.89, p ​= ​0.03, B–H adjusted p ​= ​0.09; interaction: p ​= ​0.26), driven by more negative expressions at immediate follow-up. There were no significant changes in the valence of participants’ facial expressions in response to infant vocalisations (p-values≥0.27) or toward the distressed infant video (p-values≥0.81).

### 3.6 Does the intervention enhance attention and infant-directed facial expressions toward infant stimuli?

**Fig. 4:**
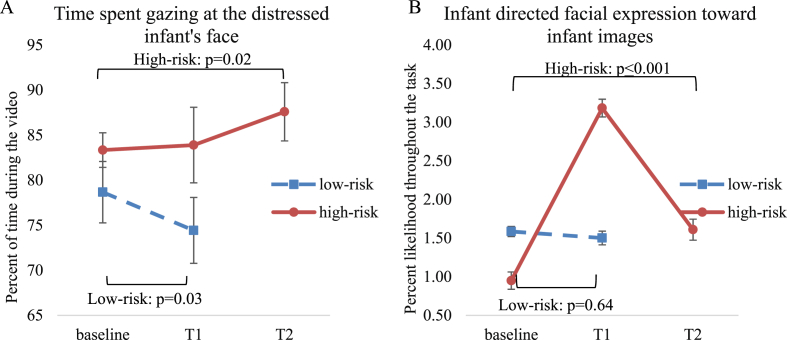
(A) High-risk pregnant women spent more time gazing at the distressed infant's face in the video at immediate follow-up (T1) and delayed follow-up (T2) compared to baseline (red solid line), while low-risk pregnant women gazed less (blue dotted line). (B) High-risk pregnant women displayed more infant-directed facial expressions toward infant face images at immediate follow-up (T1) and delayed follow-up (T2) compared to baseline (red solid line), while facial expression remained stable for low-risk pregnant women T1 (blue dotted line). Error-bars represent standard error of the mean. (For interpretation of the references to colour in this figure legend, the reader is referred to the Web version of this article.)

For the infant distress video, there was an increase over time in gaze (interaction: F(2,29.31) ​= ​4.67, p ​= ​0.02, B–H adjusted p ​= ​0.045) and fixation (time: F(2,29.45) ​= ​4.28, p ​= ​0.02, B–H adjusted p ​= ​0.07; interaction: F(2,29.25) ​= ​4.70, p ​= ​0.02, B–H adjusted p ​= ​0.03) driven by more time spent gazing at the infant at delayed follow-up than baseline (Table 4 and Fig. 4A). A trend indicated that infant-directed facial expressions toward the infant distress video were enhanced from baseline to follow-up (p ​= ​0.06).

**Table 4:** Infant directedness in high-risk pregnant participants at baseline, immediate follow-up (N ​= ​16) and delayed (N ​= ​15) follow-up.

	T0 Mean (SD)	T1 Mean (SD)	T2 Mean (SD)	T0 – T1				T0-T2			
				*F*-value (*df*_1_, *df*_2_)	*p*-value time	*p-*value interact.	d	*F*-value (*df*_1_, *df*_2_)	*p*-value time	*p-*value interact.	d
*Vigilance toward infant stimuli*											
Acc. infant vs adult	0.0 (0.7)	<0.00 (1.0)	−0.2 (0.8)	0.04 (1, 34.42)	0.84	0.40		0.13 (1.42.16)	0.72	0.70	
Acc. infant emo. vs neutral	0.5 (1.1)	0.5 (1.0)	0.6 (1.0)	0.01 (1, 42.50)	0.93	0.80		1.40 (1, 33.07)	0.25	0.11	
RT infant vs adult	27.9 (62.4)	−5.9 (50.8)	21.0 (83.7)	8.03 (1, 35.90)	0.01	0.61	0.6	0.17 (1, 31.86)	0.68	0.80	
RT infant emo. vs neutral	−3.8 (61.6)	2.4 (82.9)	20.9 (78.0)	0.07 (1, 35.00)	0.80	0.91		2.01 (1, 35.99)	0.17	0.31	
*Infant face stimuli*											
Infant-directed facial express.	0.9 (1.3)	3.2 (5.2)	1.6 (2.7)	17.53 (1, 63.56)	≤0.001	0.91	0.6	3.99 (1, 54.48)	0.05	0.82	0.3
Time spent gazing (%)	92.6 (9.0)	92.8 (6.2) ^a^	92.9 (5.0) ^b^	0.14 (1, 113.57)	0.71	0.94		0.05 (1, 111.28)	0.82	0.98	
Time spent fixating (%)	96.2 (5.7)	95.3 (5.7) ^a^	95.6 (4.2) ^b^	1.12 (1, 86.43)	0.29	0.94		0.60 (1, 85.49)	0.44	0.60	
*Infant distress video*											
Infant-directed facial express.	1.8 (2.9)	2.4 (3.07)	8.24 (15.3)	0.31 (1, 20.08)	0.58			2.47 (1, 14.02)	0.14		
Time spent gazing (%)	90.9 (13.0)	91.5 (14.3)	93.4 (10.8)	0.20 (1, 30.01)	0.66	0.97		4.48 (1, 28.20)	0.04	0.14	0.2
Time spent fixating (%)	94.5 (7.6)	93.8 (12.8)	95.4 (9.2)	0.08 (1, 30.32)	0.78	0.68		1.45 (1, 28.00)	0.24	0.25	

Abbreviations: T0, baseline; T1, immediate outcome; T2, delayed outcome; CI, confidence interval; df_1_, numerator degrees of freedom; df_2_, denominator degrees of freedom; interact., interaction; Acc., accuracy; emo., emotion; RT, response time; express, expression. *F*-values are given for the effect of time. *p*-values are listed for the main effect of time (*p*-value time) and for the emotion∗time interaction effect (*p*-value interaction). Cohens *d* specify the effect size for significant changes from T0 to T1 and from T0 to T2. ^a^ Due to technical issues, gaze and fixation for infant distress video are missing for two high-risk at T1. ^b^ At T2, gaze and fixation toward infant distress video is missing for one high-risk pregnant. Data are missing completely at random, why the missing data do not introduce bias in the results (Donders et al., 2006). We did not replace missing data points with imputation methods and therefore, analyses of the tasks with missing data were run with fewer data points.

Participants displayed more infant-directed facial expressions toward infant face images over time (infant-directed: F(2,78.59) ​= ​8.51, p ​≤ ​0.001, B–H adjusted p ​= ​0.004), driven by an increase from baseline to the immediate follow-up (Table 3 and Fig. 4B). There were no significant changes in time spent gazing (p-values≥0.51) and fixating (p-values≥0.29) toward infant face images.

For the dot-probe task, responses to infant vs. adult targets were slower at follow-up assessments than baseline (F(2, 32.07) ​= ​4.30, p ​= ​0.02, B–H-adjusted p ​= ​0.04). This was driven by slower responses at the immediate follow-up. There were no changes in RT or accuracy for other dot-probe targets (p-values≥0.23).

### 3.7 Are affective cognitive changes associated with reduction in depressive symptoms?

There were no significant changes in depressive symptoms (HDRS-17) from baseline to follow-up assessments among high-risk pregnant in the intervention group (p-values≥0.12). Affective cognitive changes did not correlate with reduction in depressive symptoms from baseline to immediate (p-values≥0.11) or to delayed follow-up (p-values≥0.28). There was a reduction in ratings of unpleasantness on the VAS from baseline to immediate follow-up (F(1, 29.52) ​= ​4.97, p ​= ​0.04), which did not correlate with affective cognitive changes (p-values≥0.08).

There was no significant change in self-reported depressive symptoms on the EPDS-scale from pregnancy to six months after birth (EPDS scores, mean ​± ​SD: pregnancy: 9 ​± ​5, post-partum: 7 ​± ​4; ​= ​0.25). Composite scores of the (i) reduction in negatively biased perception of infant stimuli, (ii) reduction in negative emotional reactivity were created and correlated with depression symptoms six months after birth. This was done by averaging z-transformed change scores from baseline to T1 and to T2 for each domain separately. These composite changes scores were created by averaging z-transformed change scores from baseline to T1 and to T2 for each domain separately. The increased sensitivity for happiness in the intervention group during pregnancy correlated with fewer depressive symptoms six months after birth (r ​= ​−0.59, p ​= ​0.03). In contrast, reduced emotional reactivity to distress in the intervention group did not correlate with depressive symptoms after birth (p ​= ​0.35).

Given the substantial variability in intervention session timelines, ranging from 8 to 27 days, we explored associations with significant changes in affective cognition. A significant correlation emerged between an increase in positive ratings of infant faces from baseline to T2 (second follow-up in pregnancy) and a shorter intervention duration (r ​= ​−0.52, p ​= ​0.049). No significant correlations were found between intervention duration and other changes in affective cognition or postpartum depressive symptoms (p-values >0.08).

### 3.8 Stability of affective cognition in low-risk pregnant

At follow-up, low-risk pregnant participants rated their own emotions in response to the most distressed vocalisations as *more negative*, they gazed and fixated significantly *less* at the infant face images and displayed *more negatively* valenced facial expressions (see Supplementary). From baseline to follow-up, ratings of emotional reactions and EDA to infant stimuli showed moderate to strong correlations, while less than half of facial expressions, gazes, and fixations and none of the dot-probe results correlated.

## 4 Discussion

This is the first proof-of-concept study of affective cognition in 16 high-risk pregnant participants before (baseline) and after (immediate and delayed follow-up) a short-term computerised affective cognitive intervention. The study demonstrated that computerised multilevel affective cognitive training is feasible and can modify attention to, interpretation of and responses to emotional infant stimuli in high-risk pregnant participants. In line with the hypotheses, the affective cognitive training (I) increased sensitivity to infant happiness, (II) reduced negative emotional reactions to infant distress, and (III) enhanced participants' infant-directed facial expressions to infant face images and attention toward infant distress. Increased sensitivity to happy infant stimuli in the intervention group during pregnancy was associated with fewer depressive symptoms six months after birth. More than half of the affective cognitive measures showed statistically significant changes in the high-risk intervention group, while only one-fifth of the measures were changed over time in the parallel low-risk control group, in support of a treatment-specific effect in the intervention group.

This novel computerised intervention proved feasible, with 80% of the included high-risk pregnant participants completing all treatment sessions and generally finding the intervention meaningful. Pregnancy is an opportune time to offer prophylactic psychological interventions as many pregnant women are interested in self-care to prepare for motherhood and want to avoid medication. The motivation for prevention strategies during pregnancy is likely greater than seeking treatment *after* birth due to stigma associated with PPD and lack of time for undergoing treatment while caring for a new-born.

The intervention improved sensitivity to infant happiness, which correlated with fewer depressive symptoms after birth. While this finding is exploratory, it provides early proof-of-concept evidence that targeting affective cognitive biases during pregnancy may attenuate the risk of depressive symptoms after birth. Increasing sensitivity in the perception of infant happiness could translate to real-life mother-infant interactions by facilitating mothers' engagement with their infants' expressions of happiness with consequent benefits for the child's psychosocial development (Mendes et al., 2014; Messinger et al., 2007; Feldman, 2003). While some degree of emotional and psychophysiological reactivity to signals of distress is adaptive, indicating a readiness for responsive caregiving, excessive negative reactivity can signal overwhelming anxiety and frustration, which is a risk factor for PPD and suboptimal mother-infant interactions. On the other hand, a ‘flat’ emotional state would impede sensitive and responsive caregiving behaviours. However, the reduction in participants' emotional reactivity and frustration toward highly distressed infant stimuli and the enhanced visual attention and infant-directed facial expressions toward infant stimuli could indicate an improvement in emotion regulation and a possible change in focus from their own emotions toward the infant. Previous research has shown that better emotion regulation ability together with more infant-oriented (vs mother-oriented) focus when listening to infant cry, predicted more sensitive maternal behaviour (Leerkes et al., 2015, Leerkes et al., 2016). By improving multiple levels of affective cognition, the current intervention may have positive implications for both the mothers' well-being, mother-infant interaction, and infant development. A previous study found that pregnant who responded more negatively to infant stimuli in pregnancy showed more adverse maternal behaviours, including intrusiveness, hostility, or flat affect responses one year postpartum (Romero et al., 2020). Therefore, it is possible that training of emotion regulation ability and sensitive infant-directedness during pregnancy may improve maternal responses to the infant after birth. While participants showed an increase in negative facial expressions in response to infant distress over time, they reported reduced frustration. This may seem counterintuitive. However, participants were instructed to mirror the infants' emotions, specifically during signs of distress. In this context, a ‘negative’ facial expression is not indicative of the participants' own emotional state, but represents a well-regulated, empathic response. It signifies a recognition of the infant's distress and an intention to comfort, aligning with effective caregiving behaviours. Thus, the increase in negative facial expressions reflects more empathic engagement and responsiveness, which are key components of sensitive parenting.

The intervention group showed no reduction in negatively biased ratings of the distressed infants’ emotions, which may be explained by the lack of negatively biased perception of infant distress at baseline. Indeed, high-risk pregnant participants perceived infant distress accurately at all assessments, in contrast with most literature that indicate negatively biased response to emotional stimuli among participants with previous depression (Bjertrup et al., 2021a; Gollan et al., 2013). Importantly, the parallel group of low-risk pregnant participants showed no changes in affective cognition with repeated testing. The intervention may have beneficial effects on psychological preparation for motherhood more broadly, if efficacy on emotional cognition and PPD risk is documented in a larger scale randomised controlled trial (RCT) that started in January 2023. Our experimental computerised assessments of negative reactivity and positive sensitivity to infants may not relate directly to how our participants respond to their own infants after birth. Nevertheless, our previous study showed an association between cognitive response to computerised infant stimuli and mother-infant interactions for mothers with affective disorders (Bjertrup et al., 2021b). However, more research is needed for optimizing and refining outcome measures to capture these nuances effectively, for example through virtual reality, and examine their ability to capture real-life mother-infant interactions.

Some limitations should be considered when interpreting the study findings. Firstly, the study had a small sample size, and no parallel control group of high-risk participants and findings should therefore only be considered proof-of-concept, preliminary and hypothesis-generating. Secondly, the immediate follow-up assessment during pregnancy took place right after the last training session, and therefore overexpose to infant stimuli may have influenced the results. In the planned RCT, the immediate follow-up of affective cognition will take place on the day after the last intensive training. Thirdly, the span of the intervention sessions fluctuated between 8 and 27 days, primarily owing to scheduling challenges. Notwithstanding this variability, subsequent post hoc analyses did not reveal any significant correlations between the duration of treatment and changes in affective cognition. Fourth, it is unknown whether the affective cognitive changes will transfer to mother-infant interaction and infant developmental outcomes or if they are limited to computerised tasks. However, a recent study showed that prenatal responses to infant cry stimuli were associated with postnatal responses to own infants (Leerkes et al., 2022), which supports the validity of computerised affective cognitive assessments. Fifth, the high-risk group was somewhat heterogenous, as both pregnant participants with previous depression and BD were included, which may explain the low sensitivity of the affective cognitive assessment to detect group differences at baseline. However, excluding BD participants from the analyses did not change the results. Sixth, the lack of baseline differences in affective cognition between high- and low-risk pregnant could indicate that the high-risk group did not have more affective cognitive risk factors for PPD than the low-risk group. On the other hand, some participants in the low-risk pregnant group could have negative cognitive biases in responses to infant stimuli that might mask any differences between our groups. In fact, we previously found that negatively biased cognitive responses to infant cries could predict PPD even in participants with no history of depression (Bjertrup et al., 2021a). Future studies should screen for affective cognitive abnormalities when including high- and low-risk participants. Seventh, it was a limitation that we did not systematically assess the effects of and potential changes in medication at every visit since this could have impacted the results. However, there were no statistically significant differences between change-score for medicated vs non-medicated high-risk participants (p-values≥0.16). Lastly, we did not analyse the influence of any other potential confounders or effect modifiers. Due to the small size of the intervention group, statistical analyses would have been underpowered for such more extensive analyses.

In conclusion, intensive two-week computerised affective cognitive training is a feasible preventive intervention strategy for pregnant at high-risk of PPD. Treatment adherence was high, the feedback was generally positive and dropouts for 20% of participants were due to non-treatment factors. The intervention was associated with improved perception of infant happiness and better regulation of emotional responses to infant distress, of which the former correlated with fewer depressive symptoms after birth. Our ongoing, larger-scale RCT will clarify whether this computerised training intervention during pregnancy can reduce the risk of PPD in high-risk individuals.

## Funding

This research did not receive any specific grant from funding agencies in the public, commercial, or not-for-profit sectors.

## Conflicts of interest

Anne J Bjertrup, Tue H Petersen, Nynne Beier, Jeanne Kofoed, Ingeborg Åse Horgen, Anette Kjærbye-Thygesen PhD and Thomas Kirkegaard reports no conflicts of interests. Kamilla W Miskowiak reports having received consultancy fees from Lundbeck and Janssen in the past three years.

## Appendix A Supplementary data

Supplementary_Bjertrup et al 2022.docx Supplementary_Bjertrup et al 2022.docx

Supplementary Table A.docx Supplementary Table A.docx
