# Supplementary material for: Prenatal affective cognitive training: A proof-of-concept study
Source: Neurosci Appl. 2023 Sep 16;2:101135. doi: 10.1016/j.nsa.2023.101135 (PMC12244145; doi:10.1016/j.nsa.2023.101135)
Supplement: Supplementary_Bjertrup et al 2022.docx [file mmc1.docx]

|  | T0 High-risk | T0 Low-risk | T1 High-risk | T1 Low-risk | T2 High-risk | T0 – T1 High-risk | | T0-T2 High-risk | |
| --- | --- | --- | --- | --- | --- | --- | --- | --- | --- |
|  |  |  |  |  |  | Parameter estimate [95% CI] | *p*-value time | Parameter estimate [95% CI] | *p*-value time |
| *VAS scores* |  |  |  |  |  |  |  |  |  |
| Happiness | 58.06 (20.48) | 70.31 (10.53) | 63.29 (15.87) | 69.48 (17.91) | 61.79 (18.72) | 5.22 [-18.36; 7.91] | 0.42 | -3.72 [-17.85;10.40] | 0.59 |
| Sadness | 35.50 (23.36) | 11.27 (15.33) | 24.43 (23.59) | 16.00 (23.52) | 17.14 (22.38) | 11.07 [-5.89; 28.04] | 0.19 | 10.50 [-6.00;27.00] | 0.20 |
| Anxiety | 31.25 (24.26) | 9.40 (14.54) | 18.93 (21.45) | 7.05 (13.35) | 17.21 (18.52) | 12.32 [-4.14; 28.79] | 0.14 | 14.04 [-1.14; 29.50] | 0.07 |
| Dizziness | 29.00 (27.81) | 7.32 (12.80) | 24.00 (22.50) | 4.67 (10.52) | 13.93 (18.93) | 5.00 [-13.15; 23.15] | 0.58 | 15.07 [-1.95; 32.09] | 0.08 |
| Nausea | 19.31 (22.00) | 16.41 (25.16) | 12.43 (17.79) | 11.00 (18.49) | 12.71 (21.64) | 6.88 [-7.47; 21.24] | 0.34 | 6.60 [-9.16; 22.35] | 0.40 |
| Alertness | 46.63 (30.51) | 46.95 (25.68) | 33.43 (27.89) | 37.14 (26.40) | 36.00 (29.26) | 13.20 [-7.84; 34.24] | 0.21 | 10.63 [-10.93; 32.18] | 0.32 |
| Unpleasantness | 46.38 (24.71) | 13.82 (20.98) | 26.93 (24.67) | 15.19 (20.40) | 28.79 (26.31) | 19.45 [1.62;37.28] | **0.03** | 17.59 [-0.90; 36.07] | 0.06 |

Supplementary Table A. Mean and standard deviations for scores on Visual Analogue Scale (VAS) of psychological and somatic symptoms in high-risk pregnant and low-risk at baseline T0, immediate T1 and delayed T2 follow-up, and parameter estimates and p-values for change scores for the high-risk pregnant intervention group.

Abbreviations: T0, baseline; T1, immediate outcome; T2, delayed outcome; CI, confidence interval. Parameter estimates are given for significant effect of time for the specific levels of emotion. *p*-values are listed for the main effect of time (*p*-value time).
