# Supplementary material for: Prenatal affective cognitive training: A proof-of-concept study
Source: Neurosci Appl. 2023 Sep 16;2:101135. doi: 10.1016/j.nsa.2023.101135 (PMC12244145; doi:10.1016/j.nsa.2023.101135)
Supplement: Supplementary Table A.docx [file mmc2.docx]

Supplementary

Methods

**Technical details on the infant-directed facial expression task and biofeedback with Affectiva**

During the *infant-directed facial expression task* participants’ faces was captured with a standard in-built webcam recording at 30 frames per second. Action Units (AU’s) of facial expressions were processed in real-time at 15 Hz (effectively processing every 2nd video frame) using the Affectiva AFFDEX 4.0 algorithm (Affectiva, Boston MA, USA). The data was stored in the background for subsequent analysis. For each videoframe processed, The Affectiva AFFDEX algorithm measured the probability of the presence of each facial expression on a 0-100% scale. Once a stimulus had ended averages of each individual facial expression probability score was calculated. In general, an average above the 10% for a given facial expression was classified as a correct response and conversely a response equal to or below 10% was classified as an incorrect response. Different facial expressions were evaluated for the different infant videos.

An adequate empathic response attuned to infant distress videos included expressions of “comfort and caring” consisting of lip pucker (AU 18), jaw drop (AU 26), inner brow raise (AU 1) and brow lower (AU 4). Specifically, participants had to display AU 1 and AU 4 and at least one of AU 18 or AU 26 for the expression to be evaluated as correct. Moreover, the facial expressions were evaluated with 15 frames, and thus 15 values for each AU on a scale from 0-100, per second in real-time. If the average of those AU values across the video was above 10%, the facial expression shown during the video was evaluated as correct. When criteria were not met, one of two written feedback is presented (in Danish): “imagine that you comfort the baby with your facial expression” or “show the baby that you understand and empathise with him/her”. Feedback, when criteria were met was: “well done, you make the baby feel better!”.

An adequate attuned response to infant laughter included expressions of “surprise, excitement, enjoyment, interest, attention, happiness, love, warmth, praise and admiration”, which included inner (AU 1) and outer (AU 2) brow raise, lip corner pull (AU 12), mouth stretch (AU 27), cheek raise (AU 6) and lips part (AU 25). Participants had to display either AU 1 or AU 2 together with AU 6 and either AU 12, AU 25 or AU 27 for the expression to be evaluated as correct. Feedback, when criteria were not met included: “return the smiles and laughter” or “try to make the baby laugh”. Feedback, when criteria were met was: “Good! You are having fun!”, “the baby is really enjoying this!” or “the smiles you display are very warm and loving”.

An adequate attuned response to infants in a relaxed, aware state included expressions of: interest, attention, happiness, love, warmth, praise, and admiration, which include inner (AU 1), and outer (AU 2) brow raise, lip corner pull (AU 12), cheek raise (AU 6) and lips part (AU 25). The pregnant women with UD had to display AU 1 or AU 2 and AU 6 and either AU 12, AU 25 for the expression to be evaluated as attuned. Feedback if the facial expressions did not met criteria included: ”pay attention to the baby’s signals” or “await the baby’s signals”. Feedback when criteria were met was: “Well done! The baby can feel your presence and interest”. Too see movements of the selected AUs in a face, see <https://imotions.com/blog/facial-action-coding-system/>.

**Explicit emotion regulation training instructions**

Instructions when watching the infant videos included: “focus friendly and curiously your attention on the baby, notice distractions, and bring back friendly the attention to the baby”, “notice and acknowledge your own inner reactions and then redirect the attention toward the infant”, “think about how the baby feels and what she/he tries to say” and ”focus on the infant with acceptance, warmth, empathy, patience and kindness”.

Instructions on loving, kind phrases for the visualisation practice included: “may my baby and I be filled with lovingkindness. May I treat myself and my baby with kindness in good times and in hard times. May I and my baby be well and live with ease”.

**Statistics, Benjamini Hochberg correction**

We adjusted for the number of analyses used to explore each of the three hypothesised affective cognitive changes. Specifically, i) negatively biased perception of infant stimuli/lowered threshold for perceiving infant happiness was investigated with two analyses, ii) negative emotional reactivity toward infant stimuli was investigated with six test and iii) enhanced infant-directedness in facial expressions and attention was investigated with eight analyses.

Results

**Stability of affective cognition in low-risk pregnant**

For the infant face images task, correlations between scores at T0 and T1 were significant for five of five ratings (r>0.56 to r<0.83, p-values< 0.01), for six of ten facial expressions (r_s_>0.46 to r_s_<0.66, p-values< 0.01) and one of ten gazes and fixations (r_s_=0.50, p=0.047) (for the remaining 13 correlations p-values>0.08).

For the infant vocalisations task, correlations were significant for nine of ten ratings (r>0.56 to r<0.90, p-values< 0.01) and for two of five facial expressions (r_s_>0.49 to r_s_<0.56, p-values<0.02) (for the remaining four correlations p-values>0.08).

For the dot-probe task, there were no significant correlations between T0 and T1 (p-values>0.17).

For the infant distress video task, the correlation between scores at T0 and T1 were significant for ratings of frustration (r_s_=0.51, p=0.02), EDA (r=0.71, p=0.001), for two of four gaze and fixation measures (r_s_>0.69 to r_s_<0.81, p-values< 0.001) and for one of two facial expressions (r=0.61, p=0.003) (for the remaining three correlations p-values>0.08).

On the infant emotional rating task, low-risk pregnant women rated their own emotions in response to the most distressed vocalisations as *more negative* at follow-up than baseline (interaction: F(4,101.53)=2.70, p=0.04, most distressed: *b*=-0.31, 95% CI [-0.50, -0.10], p=0.01; most happy: p=0.37; moderately distressed: p=0.20; moderately happy: p=0.79; neutral: p=0.56). They also gazed and fixated significantly *less* at the infant face images at follow-up than baseline (gaze: F(1,93)=9.08, p=0.03; fixation F(1,95.52)=6.49, p=0.01). However, there were no changes in ratings of the infants’ emotions (images: p-values>0.39; vocalisation: p-values>0.30) or in the valence or infant-directedness of low-risk pregnant women’s facial expressions toward infant face images (p-values>0.27). For the infant distress video, low-risk pregnant women displayed *more negatively* valenced facial expressions at follow-up than baseline (F(1,20.08)=6.99, p=0.02), but there were no changes in frustration (p=0.52), EDA (p=0.10), or in gaze and fixation (p-values>0.12). Lastly, there were no significant changes in early attention to emotional infant faces on the dot-probe task among low-risk pregnant women (p-values>0.06). A trend indicated reduction in depressive symptoms at follow-up (p=0.06). More reduction in depressive symptoms correlated with *less* reduction in gaze (r=-0,59, p=0.01) and fixation (r=-0.56, p=0.02) toward infant face images (for other correlations p-values>0.10).
